# Supplementary material for: In Vitro Anti-Inflammatory and Regenerative Effects of Autologous Conditioned Serum from Dogs with Osteoarthritis
Source: Animals (Basel). 2022 Oct 10;12(19):2717. doi: 10.3390/ani12192717 (PMC9558530; doi:10.3390/ani12192717)
Supplement: Supplementary file 1 [file animals-12-02717-s001.zip › animals-1913124-supplementary.pdf]

Table S1. For gene expression analysis, primer sequences used are as following:

Name of Genes		Primer Sequence	Amplicon Length (bp)
Collagen type I	Forward:	5′-CATCCCAGCCAAGAACTGGT-3′	139
	Reverse:	5′-GAAGGCGAGTTGAGTAGCCA-3′	
Collagen type II	Forward:	5′-CACTGCCAACGTCCAGATGA-3′	215
	Reverse:	5′-GTTTCGTGCAGCCATCCTTC-3′	
Aggrecan	Forward:	5′-ACTTCCGCTGGTCAGATGGA-3′	111
	Reverse:	5′-TCTCGTGCCAGATCATCACC-3′	
MMP-13	Forward:	5′-GGCTTAGAGGTCACTGGCAAAC-3′	118
	Reverse:	5′-TGGACCACTTGAGAGTTCGGG-3′	
Sox9	Forward:	5′-GCCGAGGAGGCCACCGAACA-3′	179
	Reverse:	5′-CCCGGCTGCACGTCGGTTTT-3′	
GAPDH	Forward:	5′-CTGAACGGGAAGCTCACTGG-3′	129
	Reverse:	5′-CGATGCCTGCTTCACTACCT-3′	
